# Little disease but lots of bites: social, urbanistic, and entomological risk factors of human exposure to *Aedes aegypti* in South Texas, U.S.

**DOI:** 10.1371/journal.pntd.0011953

**Published:** 2024-10-21

**Authors:** Nicole A. Scavo, Jose G. Juarez, Luis Fernando Chaves, Nadia A. Fernández-Santos, Ester Carbajal, Joshuah Perkin, Berlin Londono-Renteria, Gabriel L. Hamer

**Affiliations:** 1 Department of Entomology, Texas A&M University, College Station, Texas, United States of America; 2 Ecology & Evolutionary Biology, Texas A&M University, College Station, Texas, United States of America; 3 Department of Environmental and Occupational Health, School of Public Health and Department of Geography, Indiana University, Bloomington Indiana, United States of America; 4 Instituto Politecnico Nacional, Centro de Biotecnologia Genomica, Reynosa, Mexico; 5 Department of Ecology and Conservation Biology, Texas A&M University, College Station, Texas, United States of America; 6 Department of Tropical Medicine and Infectious Disease, Tulane University, New Orleans, Louisiana, United States of America; Liverpool School of Tropical Medicine, UNITED KINGDOM OF GREAT BRITAIN AND NORTHERN IRELAND

## Abstract

**Background:**

*Aedes aegypti* presence, human-vector contact rates, and *Aedes*-borne virus transmission are highly variable through time and space. The Lower Rio Grande Valley (LRGV), Texas, is one of the few regions in the U.S. where local transmission of *Aedes*-borne viruses occurs, presenting an opportunity to evaluate social, urbanistic, entomological, and mobility-based factors that modulate human exposure to *Ae*. *aegypti*.

**Methodology & Principal findings:**

Mosquitoes were collected using BG-Sentinel 2 traps during November 2021 as part of an intervention trial, with knowledge, attitudes, and practices (KAP) and housing quality surveys to gather environmental and demographic data. Human blood samples were taken from individuals and a Bitemark Assay (ELISA) was conducted to quantify human antibodies to the *Ae*. *aegypti* Nterm-34kDa salivary peptide as a measure of human exposure to bites. In total, 64 houses were surveyed with 142 blood samples collected. More than 80% of participants had knowledge of mosquito-borne diseases and believed mosquitoes to be a health risk in their community. Our best fit generalized linear mixed effects model found four fixed effects contributed significantly to explaining the variation in exposure to *Ae*. *aegypti* bites: higher annual household income, younger age, larger lot area, and higher female *Ae*. *aegypti* abundance per trap night averaged over 5 weeks prior to human blood sampling.

**Conclusions:**

Most surveyed residents recognized mosquitoes and the threat they pose to individual and public health. Urbanistic (i.e., lot size), social (i.e., income within a low-income community and age), and entomological (i.e., adult female *Ae*. *aegypti* abundance) factors modulate the risk of human exposure to *Ae*. *aegypti* bites. The use of serological biomarker assays, such as the Bitemark Assay, are valuable tools for surveillance and risk assessment of mosquito-borne disease, especially in areas like the LRGV where the transmission of target pathogens is low or intermittent.

## Author summary

*Aedes aegypti* is a mosquito vector with public health importance on the global scale as it transmits viruses such as dengue, chikungunya, and Zika. Although transmission rates of dengue and Zika are low in the U.S., there are a few regions, including south Texas, where local transmission has occurred. Our study aimed to evaluate the factors associated with risk of exposure to these viruses using a serological bioassay that measured antibody response to an *Ae*. *aegypti* salivary protein to assess human-vector contact. We collected mosquitoes, took human-blood samples, and conducted urbanistic and demographic surveys in November 2021 in eight communities in the Lower Rio Grande Valley, Texas. Our knowledge, attitude, and practices survey found that most residents recognized adult mosquitoes, though few individuals knew someone personally who been sick with a mosquito-borne disease. Outdoor adult female *Ae*. *aegypti* abundance was positively associated with exposure to mosquito bites. Household income, individual age, and lot area also significantly affected exposure levels. The Bitemark Assay we used in this study can be utilized as a tool for entomological risk assessment and could be used as an alternative to infection exposure in areas where mosquito-borne disease levels are low.

## Introduction

The yellow fever mosquito, *Aedes aegypti* L. (Diptera: Culicidae), is the main vector of arboviruses such as dengue, Zika, and chikungunya viruses. Diseases caused by these *Aedes-*borne viruses pose a threat to global health with dengue virus (DENV) affecting 390 million people annually [1], Zika having autochthonous transmission in 87 countries [2], and chikungunya causing an average annual loss 106,000 disability-adjusted life years (DALY) [3]. With a lack of safe vaccines that are effective against primary infections, public health authorities rely on controlling the mosquito vectors to reduce disease. *Aedes aegypti* vector control has shown variable levels of efficacy in reducing mosquito populations [4,5] and preventing disease transmission of arboviruses [6–8]. With the risk of a shifting global distribution of *Ae*. *aegypti* mosquitoes [9] and some regions showing potential for vector presence without associated viruses [10], novel methods of surveillance and control that focus on potential areas of arboviral emergence areas are needed.

The region known as the Lower Rio Grande Valley (LRGV) along the U.S.-Mexico border in south Texas is one of the few regions in the U.S. with local vector-borne transmission of *Aedes*-borne viruses [11], and more recently human malaria [12]. This can be explained by the fact that *Ae*. *aegypti* is well-established in the area as an efficient urban vector due to its affinity for man-made container habitat and highly anthropophilic behavior [13]. *Colonias* are low-income, mostly Hispanic communities in unincorporated areas leading to a general lack of services (e.g., poor water sanitation) [14] and marginalized residents with little political power [15]. Understanding the ecological and social factors that modulate *Ae*. *aegypti* abundance and human exposure to mosquito bites is key to developing efficient and effective vector control programs. Some of the previously detected risk factors for an increased indoor and outdoor abundance in low and middle-income communities of the LRGV include demographic indices (i.e., number of children and toddlers) and housing variables (i.e., air conditioning window-mounted units, number of windows) [16]. Furthermore, the presence of air-conditioning reduced the risk of prior exposure to dengue virus in this region [17]. More generally, household water management strategies [18], socio-economic factors (SEF) [19–22], and climate variables [23,24] have been tied to presence or abundance of *Aedes* mosquitoes. However, we currently lack the understanding of how some of these risk factors relate to human-vector interactions with the added component of human mobility or how vector abundance translates to exposure to vector bites.

Current guidelines for evaluating the success of a vector control intervention recommend the use of an epidemiological endpoint, such as active infection or past exposure to pathogens, to inform intervention efficacy [25]. However, many regions with local transmission of mosquito-borne pathogens do not have consistent and sufficient burden of human disease necessary for an epidemiological endpoint. For instance, a West Nile virus (WNV) serosurvey in humans in Connecticut, U.S. didn’t find seropositive participants in their study despite WNV being prevalent in neighboring states and that birds and mosquitoes were infected in the region [26,27]. Similarly, the LRGV represents the margin of *Aedes-*borne virus endemicity and has only sporadic local transmission of DENV (24 locally acquired cases from 2010–2017) [28]. The same pattern exists for malaria transmission, with a single autochthonous human case of malaria in South Texas in 2023 [12]. This makes evaluating an intervention using an epidemiological endpoint variable difficult to utilize in *a priori* planning of a vector control intervention study.

To overcome this limitation, the use of a human antibody response to mosquito salivary proteins has emerged as a valuable tool [29,30] to be used as a complementary endpoint which combines elements of an entomological and epidemiological endpoint. In this context, humans develop antibodies in response to exposure to salivary proteins associated with vector bites, and immunological assays can detect this past evidence of exposure to vector bites. Progress measuring human antibody response to vector bites has advanced from using crude salivary gland proteins (e.g., whole saliva) [31,32] to more recent progress identifying peptides specific to different mosquito taxa, improving assay specificity [33,34]. Moreover, such tools can be used to estimate the risk of arbovirus exposure in areas with high transmission [33,35] and are a more direct measure of risk than adult female mosquito abundance. Although adult female abundance has been correlated with dengue incidence in some cases [36,37], there are some limitations to this approach. For instance, human landing catches cannot be ethically performed with *Aedes* species [38] as there are no universally effective vaccines for *Aedes-*borne viruses, except for yellow fever virus. Because of this, mosquito sampling of host-seeking, oviposition seeking, or resting populations are used but these do not measure mosquito contact with humans and thus do not account for mosquito or host behaviors (e.g., use of spatial or personal repellents), and which have different levels of efficiency [39].

It has been shown that for different mosquito species the use of IgG antibody response to mosquito salivary gland proteins can serve as effective indicators of human-vector contact as an exposure biomarker [34,40–42]. Moreover, high bite exposure measured via this method has been linked to disease levels in humans for both the malaria [34,40] and dengue systems [33,43] indicating its usefulness in assessing mosquito-borne disease risk. IgG antibodies are used over IgM as their specificity is greater [33], and is specific to the genus level [44], with low levels of cross-reactivity between *Ae*. *aegypti* and *Ae*. *albopictus* [45].

Even so, immune response intensity is not always linked to the likelihood of being bitten by infected mosquitoes [38]. Although a higher immune response likely means more bite exposure, it might not directly translate to be being bitten by a mosquito that is positive for dengue. In Fustec et al. [38], the direct relationship of human dengue infections and antibody response to *Aedes* salivary proteins could not be measured as no dengue cases were detected during the study period [38], and other studies have shown a link between exposure levels and dengue cases in humans [33,43]. Salivary biomarkers also allow for individual mosquito bite exposure assessment and improve the ability to assess heterogeneity of disease transmission compared to community-level entomological measures [42].

Our study focuses on comparing the use of an antibody response against *Ae*. *aegypti* salivary gland peptides (i.e., Nterm34kDa) as an endpoint measurement in relation to mosquito abundance. Also, we aim to describe social, urbanistic, and human mobility risk factors associated with *Ae*. *aegypti* abundance and exposure to their bites in low-income communities (a.k.a. *colonias*) of the LRGV. The results build on our previous work in the area to elucidate seasonal patterns of mosquito abundance [46], dispersal of *Ae*. *aegypti* from discarded containers [47], and evaluating vector control interventions [5]. Ultimately, our work can help guide public health programs to better understand the local ecology of mosquitoes along the U.S.-Mexico border and how vector control interventions might be evaluated in the LRGV and in other regions.

## Methods

### Ethics statement

This project received approval from the Institutional Review Board of Texas A&M University (IRB2021-0886D). We obtained individual written consent from each household owner for the weekly outdoor entomological surveillance and KAP surveys. We obtained individual written consent from adults that participated in the blood sampling and assent from children, as well as written consent from a guardian, for the same procedure.

### Study location and site selection

The study was carried out in the county of Hidalgo, Texas, U.S., which is part of the LRGV region located along the U.S.-Mexico border. The county of Hidalgo has an estimated 870,000 inhabitants, of which 92% consider themselves Hispanic or Latino origin, 26% are foreign borne individuals and 24% live in poverty (based on income and family size/composition) [48]. The climate in this region is considered humid sub-tropical, with a cold/dry season from November to February (7–21°C), and a rainy season that starts in April (18–30°C), peaks in September (23–33°C) and finishes in October (19–31°C) [49].

**Fig 1:**
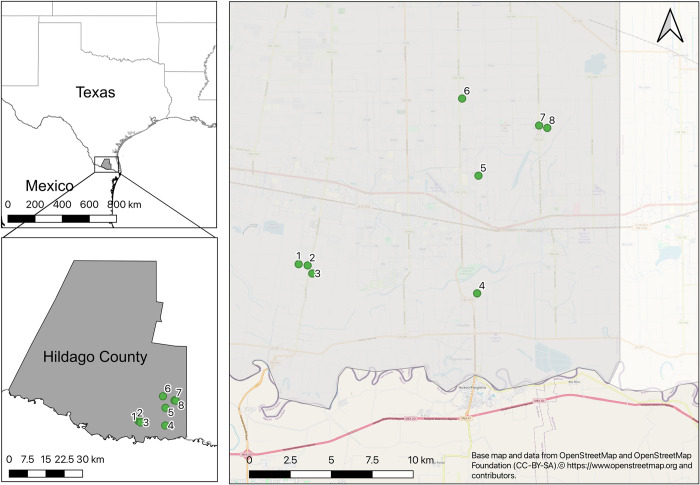
Study sites in Hidalgo County in south Texas. 1 –La Piñata, 2 –South Donna, 3 –Balli, 4 –Progresso, 5 –Chapa, 6 –Mesquite, 7 –Indian Hills West, 8 –Indian Hills East. County and state boundaries from US Census Bureau’s Cartographic Boundary Files (https://www.census.gov/geographies/mapping-files/time-series/geo/carto-boundary-file.html) and the base map is the OpenStreetMap which is available under the Open Database License (https://tile.openstreetmap.org/{z}/{x}/{y}.png).

Sites were selected based on previous work in the area [16,46] where rapport had been built with community members. Briefly, potential sites were selected based on average income level per household, total number of households in the community, isolation of community, and distance from our base of operations in Weslaco, Texas. We selected eight low-income communities based on high community participation in past studies (Fig 1).

### Entomological sampling

Adult mosquito sampling was carried out as part of an Auto-Dissemination Station (ADS) (BanfieldBio Inc.) intervention study, similar to a previous trail using a different ADS prototype [50]. The intervention study was a cluster randomized control trial that was carried out from June 2021 until March 2022. Surveillance was not conducted for an entire year (52 weeks) given that the risk of *Aedes*-borne virus transmission such as DENV and ZIKV in the LRGV peaks from September to November when the *Ae*. *ageypti* is most abundant, this is common practice in the area [51,52]. Mosquito sampling was done using BG Sentinel 2 traps (Biogents, Germany) baited with BG lures (Biogents, Germany) placed in the peridomicile (i.e., outside) of homes at a density of 1 trap per 500m^2^, with a up to 84 traps set per week, over 39 weeks. A total of 89 household participated at some point in the mosquito sampling. Traps were left for 24 hours once per week, collected mosquitoes were separated by sex and species (*Ae*. *aegypti*, *Ae*. *albopictus*, *Culex* sp., *Anopheles*, and other mosquitoes) and stored at -20°C. Mosquito identification was done based on morphology using taxonomic keys [53]. Households within communities were randomly selected based on desired trap density. Houses were approached for participation in the study and if homeowners agreed, a trap was placed in their lot. If a household dropped out of the study, a neighbor was recruited as a replacement in the following order: neighbor to the right, neighbor to the left, neighbor in behind, neighbor directly across the street.

### Surveys and blood sample collection

Knowledge, Attitudes, and Practice (KAP) and housing quality surveys were conducted concurrently at the same household visit from November 1–13, 2021. The format of the surveys was similar to those previously done by our group in 2017 and 2018 [16]. Briefly, we used a structured face-to-face questionnaire with a mixture of close-ended, semi-closed-ended, open-ended and ranking questions. The KAP survey asked participants about household demographics, mosquitoes, the pathogens mosquitoes transmit, and their household member movement patterns. The house quality survey consisted of evaluating the quality of windows, doors, and their screens; housing construction material; and water holding containers and was carried out counterclockwise from the main house entrance. We recorded housing materials (timber/metal, cement, brick), screen quality (with holes, with no holes, size of holes) on windows and doors, the type of air conditioning (A/C) unit (window mounted, central) if present, and the type and number of mosquito container habitats found in each household peridomicile (see S1 Data). We defined the peridomicile of a household as the area between the property limit to the main house perimeter. From 95 houses under weekly entomological surveillance, 47 agreed to participate with the KAP, blood sample and housing surveys. An additional 17 houses were recruited as replacements (replacement houses were recruited from the left, right, back and front, adjacent to the BG house, if no house agreed no further house was recruited) for a total of 64 houses surveyed.

Blood sampling was done via finger prick and four circles of blood were collected on a 903-protien saver card (GE Healthcare, USA). Samples were dried and placed into a plastic bag with desiccant and stored at 4°C until further processing.

### Bitemark assay

The *An*. *gambiae* gSG6-P1 peptide for the measure of exposure to *Anopheles* spp. [34] and the *Ae*. *aegypti* peptide Nterm-34kDa [54] were synthesized by Genscript (Piscataway, NJ, USA). ELISA conditions were standardized as described elsewhere [34,43]. Briefly, dried blood samples were prepared by punching a 6mm circle out of the Whatman 903 protein saver card (GE Healthcare, US), and eluting it into 500 μL of elution buffer (PBS 1×) and incubating overnight at 4°C. At the time of sample preparation UltraCruz High Binding ELISA Multiwell Microplates (96-well) were coated with 100 μL/well of either gSG6-P1 or Nterm-34kDa peptide (2 μg/mL). Plates were incubated overnight at 4°C and blocked with 200 μL of 5% skim milk solution in PBS-tween 20 (0.05%) (Blocking buffer) for 30 min at 37°C. The sample elution was used to prepare a 1:50 dilution of the sample in blocking buffer. Then, 100 μL of that dilution were added to each well (individual samples were tested in duplicate). Plates were incubated at 37°C for 2 h, washed three times, then incubated 1 h at 37°C with 100 μL/well of a 1/1000 dilution of goat monoclonal anti-human IgG conjugated with horseradish peroxidase (ABCAM, Cambridge, MA). After three final washes, colorimetric development was carried out using tetra-methyl-benzidine (Abcam) as a substrate. In parallel, each assessed microplate contained in duplicate: a positive control (pool of diluted samples), a negative control (wells with no human sample), and a blank (Wells with no antigen). The blank was composed by wells containing no sample. The reaction was stopped with 0.25 N sulfuric acid, and the optical density (OD) was measured at 450 nm.

Optical density normalization and plate to plate variation was performed as previously described by our group and others [34]. Briefly, antibody levels were expressed as the ΔOD value: ΔOD  =  ODx − ODb, where ODx represents the mean of individual OD in both antigen wells and ODb the mean of the blank wells. For each tested peptide, positive controls of each plate were averaged and divided by the average of the ODx of the positive control for each plate to obtain a normalization factor for each plate as previously described [34]. Each plate normalization factor was multiplied by plate sample ΔOD to obtain normalized ΔOD that were used in statistical analyses.

### Statistical analysis

We collected 99 variables between the KAP and housing surveys. To make the analysis more manageable, we used dimension reduction methods to generate three indices (windows, doors, and hosts) following the procedures described in Chaves et al. [55]. To start, we carried out descriptive statistics on the KAP and housing datasets to assess which variables had low standard deviation which would impact the results of our data reduction techniques. We removed variables that fell under these categories, that were colinear in nature, or that had a high degree of missing values not collected in the original surveys. The door and window indices were generated via Principal Components Analysis (PCA) by grouping variables according to their relevance to door, window, and host categories. Other indices were tested, though only these three were kept as they explained more than 50% of the cumulative variability. Details on index creation and PCAs biplots can be found in S1 Statistical Analysis.

After data reduction and elimination of identification variables (e.g., street name, latitude, longitude) from the dataset, 47 explanatory variables remained. We then chose to select 10 variables from this set so that our *n*/*k* value, where *n* is the number of data points and *k* is number of parameters, would be above ten [56]. We used our knowledge of the literature to identify 10 variables from our set that were relevant to our study [56,57]. Rows with missing data were omitted from the analysis.

Our outcome variable of interest was individual exposure to *Ae*. *aegypti* bites measured via the Bitemark Assay (i.e., ΔOD). We analyzed how social, urbanistic, entomological, and movement factors were associated with bite exposure using a generalized linear mixed model (GLMM) approach. A mixed model was chosen to account for the potential lack of spatial independence (i.e., individuals nested within households and households nested within communities). While both linear mixed models [38,58] and GLMMs [59–61] have been used in similar serological-biomarker studies, we chose a Gaussian GLMM because they are an ideal tool for analyzing normal data whose independence is constrained by different factors (e.g., spatial or temporal) and that are modeled as random effects [62,63] in our study.

We constructed a global model (mglobal1B) to evaluate the effect of 10 fixed effects on ΔOD while controlling for non-independence among houses surveyed in the same communities. The 10 fixed effected included average distance in miles traveled per week, income (2 levels: <$25,000, >25,000), host community index (host.1), door index (door.2), age (years), sex (2 levels: male, female), AC type (4 levels, window, central, mini-split, none), average abundance of *Ae*. *aegypti* females averaged over 5 weeks prior to sampling, area of lot (m^2^), and total containers in the lot (Table A in S1 Statistical Analysis). Details on variable selection can be found in S1 Statistical Analysis. Individuals nested with homes with homes nested within each of eight communities was set as a random effect. Restricted Maximum likelihood (REML) was used due to the unbalanced nature of our dataset [62]. Abundance of *Ae*. *aegypti* females was averaged over 5 weeks as IgG response to bites has been shown to appear between 1 to 6 weeks post exposure [30], and to specifically last for a least 4 weeks in *Aedes* [64], and to be more persistent than *Anopheles* IgG responses [65].

We first created a global model (mgloabl1B) that had a Gaussian distribution with an identity link as our outcome variable is continuous. To check model assumptions, we plotted the distribution of residuals and assessed QQ plots. Results showed deviation from the expected distribution, so we next ran another global model (mglobal2B) using a log link for a Gaussian distribution. Plotted residuals from mglobal2 showed a normal distribution. Backward elimination was used to simplify mglobal2B such that simpler models with lower AIC values were kept [57,66].

In addition, we were interested in analyzing the risk factors that modulated *Ae*. *aegypti* female abundance, to compare results to out prior study in the same region with independent data [16]. We used a similar approach to our analysis of the Bitemark Assay, using a GLMM to assess different factors that were indicated previously important by a review of the literature. Again, we constructed a global model (mgloabl1A) that included 10 explanatory fixed effects: presence of water storage devices in the yard, total number of containers, host index 1, host index 2, income, vegetation level (i.e., percentage of yard coverage by vegetation) area in m^2^, orderliness of yard, precipitation, and maximum temperature. Random effects were community and intervention arm. First, we modeled a Poisson error distribution in which variance = mean and compared this to negative binomial distributions (type 1and 2) in which variance > mean [67,68]. Once the correct distribution was chosen based on evaluation of QQ plots and AIC values backward elimination was again used to simplify the models and evaluated based on AIC values. All models were generated, and figures were created using R Version 4.3.2 (September 1, 2023), except for Fig 1 which was created using QGIS (version 3.16.6-Hannover). R code can be found in S1 Code and a more detailed statistical description can be found in S1 Statistical Analysis.

## Results

In total, 64 adult humans from different households were interviewed using our KAP survey. The human knowledge level of adult mosquitoes was high with 100% of interviewees recognizing an adult mosquito specimen. Fewer individuals were able to identify larval or pupal mosquitoes (35.9%). Most interviewees (90.6%) believed mosquitoes affect their families either as a nuisance, health risk, or cause of allergies, though the level of the problem they believed mosquitoes caused varied (Table 1). Knowledge of mosquito-borne diseases was also high, 81.3% of respondents had heard of them before the interview. Most participants (82.8%) considered mosquito-borne disease a risk to their community, though few knew someone personally who had been infected with a mosquito-borne pathogen (28.1%). Lots surveyed had an average size of 682 m^2^ (sd = 236) with a variety of vegetation cover and vegetation height. Few lots (14.3%) actively stored water on their property for later use. However, most lots (76.6%) had other containers that could serve as larval habitat if filled with water via a rain event. Most houses had some form of air conditioning with window units being the most common followed by central systems (Table 2).

**Table 1:** Knowledge, attitudes, and practices of household heads in the Lower Rio Grande Valley, Texas related to mosquitoes and their diseases.

Knowledge, Attitudes, & Practices	Response	No. positive responses/Total (%)
Mosquitoes	Recognized mosquito larvae or pupae	23/64 (35.9)
	Recognized adult mosquitoes	64/64 (100)
	Believed mosquitoes effect their families	58/64 (90.6)
	Believed mosquitoes were most abundant in the summer	53/64 (82.8)
	Believed mosquitoes were most abundant in the evening	57/64 (89.1)
	Considered mosquitoes to be a problem in their community Small or moderate problem Serious problem Very serious problem	62/63 (98.4) 28/63 (44.4) 15/63 (23.8) 19/63 (30.1)
Mosquito-borne diseases	Had heard about mosquito-borne diseases Dengue Zika Chikungunya West Nile virus Malaria	52/64 (81.3) 31/64 (48.4) 28/64 (43.8) 5/64 (7.8) 4/64 (6.3) 3/64 (4.7)
	Considered mosquito-borne diseases of concern to their community Low Moderate High	53/64 (82.8) 11/64 (17.2) 14/64 (21.9) 27/64 (42.2)
	Knew someone who had been infected with a mosquito-borne disease	18/64 (28.1)

**Table 2:** Housing and lot variables in the Lower Rio Grande Valley.

Question	Response	No. positive responses/total (%)
Water storage on property	Yes	9/63 (14.3)
Air conditioning type	None Window Minisplit Central	3/63 (4.8) 39/63 (61.9) 6/63 (9.5) 15/63 (23.8)
% cover of vegetation in lot	< 25 25–50 51–75 > 75	20/64 (31.3) 14/64 (21.9) 22/64 (34.4) 8/64 (12.5)
Vegetation height	< 5 cm > 5 cm	37/64 (57.8) 27/64 (42.2)
Level of shade cover	None Little A lot	7/64 (10.9) 34/64 (53.1) 23/64 (35.9)
Orderliness	Disorderly Average Orderly	25/64 (39.1) 25/64 (39.1) 14/64 (21.9)
Housing type	Custom Manufactured Mobile	27/63 (42.9) 17/63 (27.0) 19/63 (30.2)
Roof material	Shingles Metal Other	48/63 (76.2) 10/63 (15.9) 5/63 (7.9)
Wall material	Brick Cement Timber Other	8/64 (12.5) 12/64 (18.8) 39/64 (60.9) 5/64 (7.8)
Larval containers	Absent Present	15/64 (23.4) 49/64 (76.6)

Over the course of the five weeks before the blood samples were collected, a total of 1,379 female *Ae*. *aegypti* were collected, with an average of 2.9 ± 0.005 caught per trap day. The mean of ΔOD values from the Bitemark Assay for *Ae*. *aegypti* was 0.12, with a range of 0.05–0.43. A total of 187 female *Ae*. *albopictus* were found during the five weeks prior to blood samples, with an average of 0.26 caught per trap day. No *Anopheles* were caught during the five weeks prior to human blood sampling. The range of ΔOD for *Anopheles* was 0.08–0.35 with a mean of 0.18 ± 0.004.

**Fig 2:**
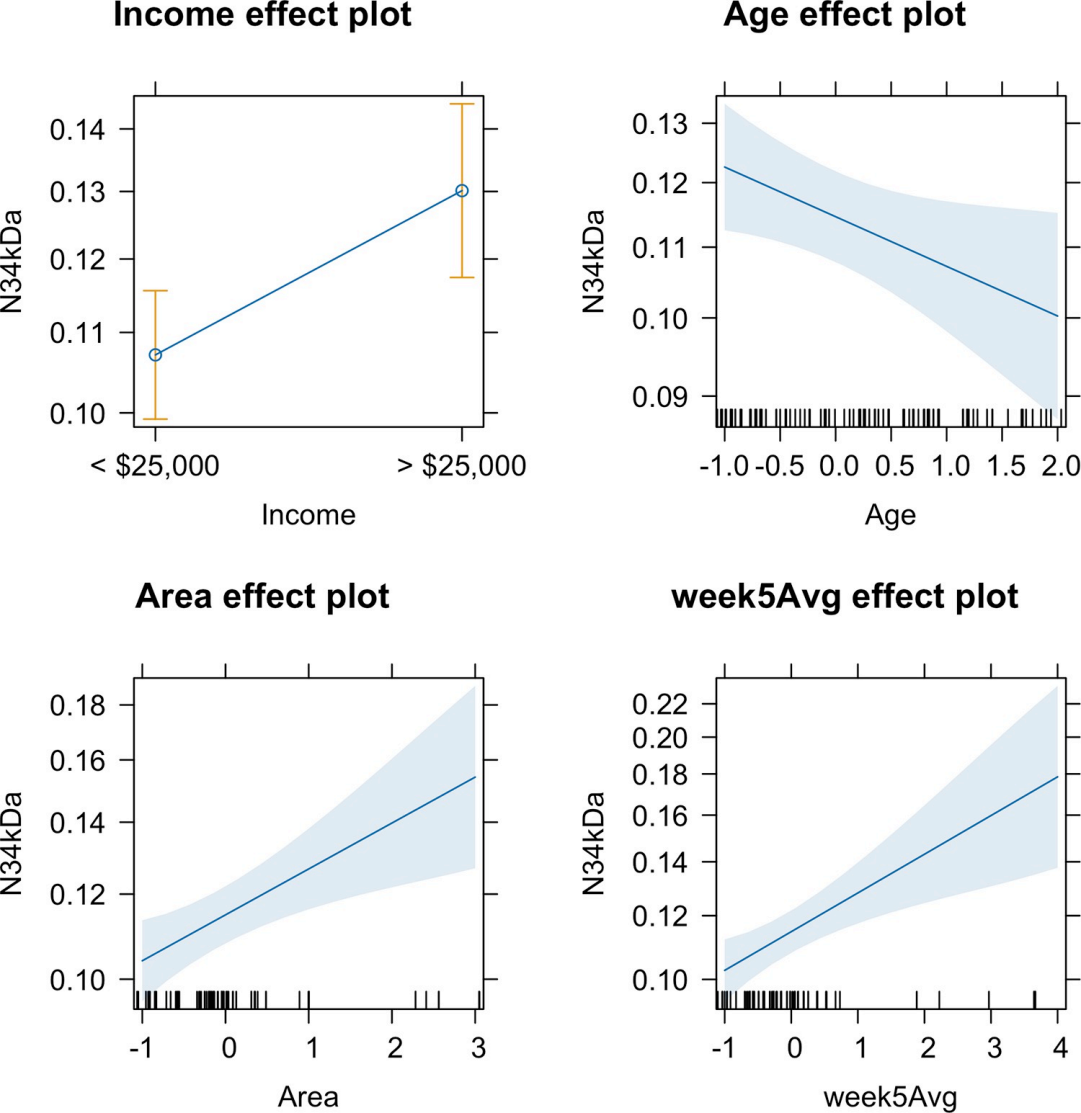
Effect plots of the best fit GLMM for human exposure to *Ae*. *aegypti* bites. N34kDa indicates antibody levels, income is represented in U.S. dollars, age was measured in years and was standardized, areas measured in m^2^ and was standardized, and week5Avg is the average number of female *Ae*. *aegypti* adults caught per trap night in the 5 weeks prior to sampling.

Given the large number of variables we collected, three indices were generated by grouping variables of similar nature together using PCA: door, host, and window. Plots of the PCAs and their interpretation can be found in S1 Statistical Analysis. Descriptive statistics of the explanatory variables can be found in Table A in S1 Statistical Analysis. Minimization of AIC via backward elimination identified the best fit model which considered the following covariates: annual household income, age of participant, lot area, and the female *Ae*. *aegypti* abundance per trap night averaged over five weeks prior to human blood sampling. Households with an annual income of >$25,000 (i.e., 35% of households) were 1.21(Exponentiated 95% CI: 1.06–1.39) times more likely to be exposed to *Ae*. *aegypti* bites. Age was treated as a continuous variable and was also a significant indicator of bite exposure, with older individuals being 7% less likely to be bitten for each year of age. Larger lots were 1.11(Exponentiated 95% CI: 1.04–1.17) times more likely per m^2^ to have individuals in the household exposed to bites. For each additional adult female *Ae*. *aegypti* in the lot, humans were 1.12 (Exponentiated 95% CI: 1.05–1.18) times more likely to be exposed to bites. Individual effects of each predictor variable can be seen in Fig 2.

**Fig 3:**
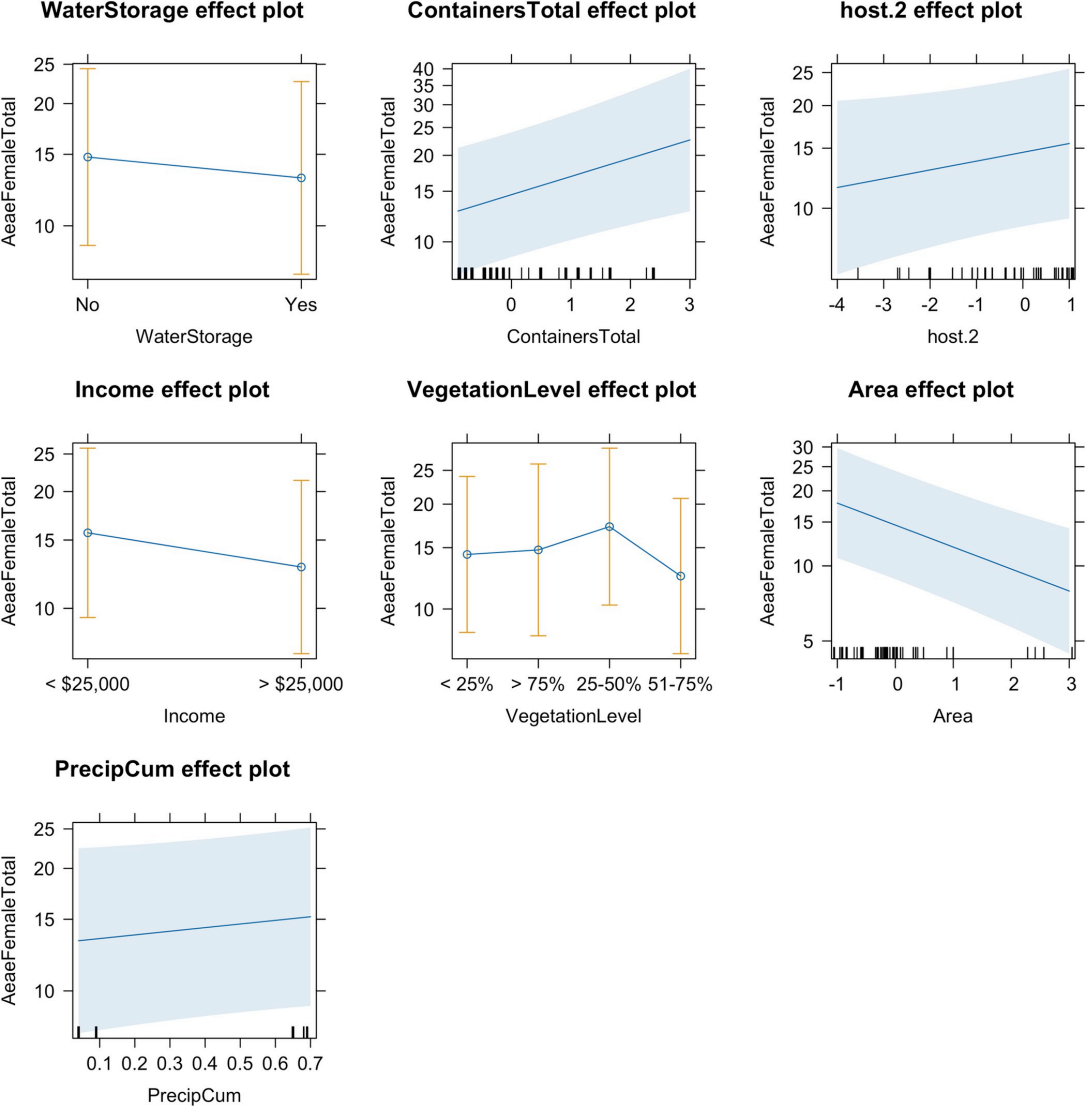
Effect plots of the best fit GLMM for adult female *Ae*. *aegypti* abundance.

Additionally, we analyzed factors that were associated with female *Ae*. *aegypti* abundance in the yards of sampling households. Evaluation of QQ plots and minimization of AIC values identified the Negative Binomial 2 distribution as the best fit. Backward elimination and assessment of AIC values identified the best fit model (m2A) that included the following variables: water storage in the yard, total number of containers in the yard, host index 2, annual household income, vegetation level in yard, area in m^2^, and cumulative precipitation in inches. Four of these variables were statistically significant. Unsurprisingly, more water-holding containers in the yard led to more female *Ae*. *aegypti* with each additional container leading to 1.16 (Exponentiated 95% CI: 1.06–1.27) increase in mosquitoes. Area had an positive relationship to mosquito abundance, with smaller yards having 18% fewer mosquitoes than larger yard (Exponentiated 95% CI: 0.74–0.90). Higher income (i.e., >$25,000) was associated with lower relative abundance of *Ae*. *aegypti* females. And lastly, medium levels of vegetation in the yard had a positive relationship with the number of females in the yard. Individual effects of predictor variables can be seen in Fig 3.

## Discussion

The results from our KAP survey indicate that most residents recognized adult mosquitoes, had heard of mosquito-borne disease, and considered it to be a problem in their communities. Even so, less than one third of residents surveyed knew someone personally who had been affected by a mosquito-borne disease. These results further illustrate that the LRGV is an area of low dengue endemicity [10] where human disease outcome variables such as human dengue incidence are not good surveillance or intervention evaluation tools. In this context, measuring human antibody response to species-specific mosquito salivary proteins (i.e., salivary biomarkers) is a good tool to use in areas like the LRGV where infection risk is comparatively low but still present [69].

We evaluated the Bitemark Assay, which measures to IgG response to the *Ae*. *aegypti* Nterm-34KDa peptide, to relate female *Ae*. *aegypti* abundance to human exposure to their bites with the goal of evaluating a complementary tool in the surveillance of *Ae*. *aegypti*, their associated viruses, and interventions aimed at *Ae*. *aegypti* population control. Since serological biomarkers have been suggested as a cheaper and quicker option than more traditional entomological measures (e.g., larval or adult abundance indices) [69], it is important to assess the relationship between bite exposure and *Ae*. *aegypti* abundance. Our results corroborate a previously established link between *Ae*. *aegypti* abundance and bite exposure measured via a serological biomarker, a link that has been shown for both larval [59] and adult [38] abundance measures. Humans in homes with higher abundance of *Ae*. *aegypti* in traps had 1.12 times higher exposure for each individual mosquito than humans in homes with fewer *Ae*. *aegypti* in traps. We did not sample the indoor vector abundance as we have done in past studies in these communities [46], so we don’t know how this positive but weak association with outdoor abundance would have compared to indoor abundance. In a previous study, a positive relationship has been shown between mosquito density, both indoors and outdoors, and bite exposure [38]. However, several studies on *Aedes* salivary biomarker’s link to density are either focused on immature mosquito life stages or do not specify the location of trapping, making it difficult to make comparisons to our work [38,44].

Our results indicate that there are other factors–environmental and social in nature—that predict exposure to *Ae*. *aegypti* bites in the LRGV. Socially, age and income were significant predictors of exposure. A higher income (i.e., >$25,000), led to more exposure to *Ae*. *aegypti* bites, a finding that is an expected extension of Juarez et al. [16] work in the area who found that medium income, i.e., $25,000–50,000, was an indicator of higher outdoor *Ae*. *aegypti* relative abundance than in low- or high-income areas. While Martin and colleagues [46] found low-income communities to have a higher relative abundance of *Ae*. *aegypti* mosquitoes than mid- or high-income communities, their study design drew income data from the U.S. Census at the block level where our study and Juarez et al. [16] used income data at the household level. These differences in scale could explain the difference in the results, a phenomenon widely described in ecology as the paradox of how resource availability is described depending on a density measurement [70]. Another possible explanation for these differences is that all our communities are classified as low-income and it is possible that higher income households, within this lower income context, have more exposure to *Ae*. *aegypti* bites. This could be due to these households have more resources for outdoor spaces such as more plant pots-saucers or water features that could serve as habitat for larva. Moreover, the relationship between socio-economic factors, such as income, and *Aedes* mosquitoes is variable and often determined by geographical context, with a slight majority (50–60%) of studies showing greater mosquito abundance in areas with lower socio-economic status (SES) [71]. Lastly, it is important to point out that exposure to bites is not a proxy for adult female abundance. Though we observed a positive relationship in between exposure and adult female abundance, our results indicate that they are modulated by different factors and should not be used interchangeably.

To the best of our knowledge, our study is the first to find a relationship between SES, as measured by income, and exposure to *Ae*. *aegypti* bites. Other studies have showed mixed results in terms of SES variables and bite exposure. For example, one SES variable, occupation type, has been associated with exposure previously [38] while education level has been shown to have no effect on exposure [60]. Lastly, it is worth noting that the effect of income on exposure to *Aedes* bites was small but significant, which likely means it explains a small proportion of the variation in the data. Confounding variables, such as mobility, where individuals spend most of the day (e.g., indoors, outdoors, at school), and indoor abundance of *Ae*. *aegypti*, that were not directly measured in this study or were not included in the models may be playing a key role. This caveat should also be considered for the effect of age on exposure to *Aedes* bites.

In our study, age was negatively associated with exposure to *Ae*. *aegypti* bites indicating that younger people were more likely to be bitten than older people. In previous studies, age has been a contributor to bite exposure levels, though the directionality of the relationship varied among studies [38,59,60]. Doucoure and colleagues [44] propose three hypotheses to understand the difference in exposure between adults and children: 1) antibody response is directly correlated to the bites received, 2) children have stronger reactions to bites than adults, or 3) adults experience desensitization to bites. Although this study was not designed to interrogate these three hypotheses, we observed that children had different mobility and behaviors than adults, such that they spent most of their time away from home (i.e., at school) or inside (i.e., sleeping). These differences could translate to different exposure levels to *Ae*. *aegypti* bites (first hypothesis). It is worth noting that human attractiveness to mosquitoes is highly variable, and can be influenced by factors including skin microbiota, diet, pathogen infection status, and genetics [72]. Specifically, smaller individuals, such as children, may produce less carbon dioxide or volatile chemicals to attract mosquitoes. Our results showed consistency with previous work in regard to lot size [16,73], i.e., larger lots were associated with higher bite exposure. We did not find any association between bite exposure and human mobility, even though previous modelling [74–76] and empirical studies [77] have shown a relationship between mobility and dengue virus transmission. While our results were unexpected, it is important to note that we asked participants about their movement patterns during the interview portion, which could have led to inaccuracies due to memory lapse. Moreover, mobility may affect dengue transmission dynamics as mentioned above, but not bite exposure as *Ae*. *aegypti* travels only within a 200m area [47] compared to the kilometers that humans move [72].

Serological biomarkers are valuable tools in a variety of contexts related to arthropod vectors, the pathogens they transmit, and their control. Here we presented a study showing the relationship between the serological biomarker, *Aedes* Nterm-34kDa peptide, and adult female *Ae*. *aegypti* abundance or density as well associated risk factors for bite exposure. An additional application of this tool is the evaluation of vector control interventions. Serological biomarkers, such as gSg6-PI and cE5, have been used to effectively evaluate *Anopheles* and malaria interventions [31, 32, 78] and the Nterm-34kDa peptide has also been used to effectively evaluate *Ae*. *albopictus* [64]. Though, to our knowledge, there has not been use of serological biomarkers to evaluate interventions of *Ae*. *aegypti*, this study and another similar study in Thailand [38] were conducted as part of intervention studies that may have yet to be published.

In addition to assessing risk factors related to bite exposure, we investigated how environmental and social risk factors affected adult female *Ae*. *aegypti* abundance. The total number of containers in the yard was positively associated with adult female *Ae*. *aegypti* abundance. These results seem intuitive, it is important to remember that the type of container can affect its production level [79] as well as the other environmental variables such as water volume, presence of nearby trees, and water temperature [80]. Previously in the LRGV, more tires in a yard led to fewer female mosquitoes in the yard, [16] which may indicate the type of container is an important factor determining productivity in the LRGV. Larger yards and lower annual household income were also associated with more mosquitoes. Lastly, medium levels of vegetation in the yard were associated with more adult mosquitoes.

Our results show that different factors explain the variation in adult mosquito abundance and exposure to their bites. Our best fit model for describing adult abundance showed that the number of containers in the yard, the size of the yard, the vegetation level in the yard, and the household income modulated abundance, but this model only explained 54% of the variation in the data when the random effects were included (conditional R^2^ = 0.54). So, it is unsurprising that the models do not match well as they explain a small amount of the variation in the data. Moreover, the best fit models for bite exposure and adult abundance were based on different explanatory models which could also explain the differences in the models. These results suggest that serological biomarkers are not a direct proxy for arthropod vector abundance. The two measure represent different things and are modulated by different factors. Given momentum of the field utilizing serological evidence of human and animal exposure to vector bites, more research should investigate how vector abundance relates to vector bites.

Our study has several limitations. First, our group has been working in the LRGV in the same communities for at least 5 years. Because of this, the people we surveyed may have more knowledge about mosquitoes and their biology compared to the wider community since they have had years of exposure to our past community engagement and outreach [81]. Moreover, most of our surveys were conducted during the weekday, meaning that generally retired or individuals without normal business hour work schedules were surveyed. This could have biased the age structure of our study. Additionally, *Ae*. *albopictus* are present in the sampling communities and some were caught in our BG traps during the study period. While the Nterm-34kDa serological biomarker is considered specific to the species level [44], cross-reactivity between *Ae*. *aegypti* and *Ae*. *albopictus* has been observed [45, 64]. Though this could confound the outcomes of our study, *Ae*. *aegypti* are more abundant in the area and since *Ae*. *albopictus* still pose a risk for *Aedes-*borne arboviruses, this information is still relevant to the control of their spread.

Our study supports the use of the Nterm-34kDa serological biomarker as a proxy for adult *Ae*. *aegypti* entomological surveillance as we found a significant, though weak, relationship between exposure to *Ae*. *aegypti* bites and adult female abundance. This relationship is consistent with other studies and in our context, demonstrates serological biomarkers as a valuable tool to use as a proxy for adult female abundance or in areas where transmission of *Aedes-*borne pathogens is low, such as the LRGV. Additionally, we recommend the use of this tool in risk assessments which can complement more traditional entomological measures as it is a more direct measure of exposure to bites than female abundance alone. In similar studies in the future, we recommend better resolution of human mobility such as the use of GPS units [82] or smart phone apps [83] to improve the relationships of this important variable to vector exposure. And finally, we suggest further studies on the use of the Bitemark Assay to provide insights into the strength of this tool to measure risk factors associated with human exposure to vector bites as well as an outcome variable for vector control trials.

## Supporting information

S1 Statistical Analysis Detailed statistical analysis with R code excerpts included. (DOCX)

S1 Code R code for the statistical analysis. (RTF)

S1 Data Database. (XLSX)
