# Supplementary material for: Little disease but lots of bites: social, urbanistic, and entomological risk factors of human exposure to Aedes aegypti in South Texas, U.S
Source: PLoS Negl Trop Dis. 2024 Oct 21;18(10):e0011953. doi: 10.1371/journal.pntd.0011953 (PMC11527178; doi:10.1371/journal.pntd.0011953)
Supplement: S1 Statistical Analysis — (DOCX) [file pntd.0011953.s001.docx]

Bitemark Analysis

## Supplementary Material 1: Statistical Analysis

This supplementary document outlines our statistical analysis for risk factors associated with exposure to *Aedes aegypti* bites and for social and urbanistic variables that are associated with female *Ae. aegypti* relative abundance in the Rio Grande Valley, Texas, USA. We used generalized linear mixed effects models (GLMM) for these analyses. Our large dataset was reduced using Principal Component Analysis (PCA) to create three indices and predictor variables were chosen based on our knowledge of the literature. The best fit models were selected based on lowest Akaike information criterion (AIC).

## Data Reduction via PCA

A large number of variables were collected (99) through Knowledge, Attitude, and Practices (KAP) and housing surveys. We used PCA to create three indices - door, window, and hosts - by grouping variables that were similar in nature. First, we conducted descriptive statistics to determine which variables had low standard deviation which would impact the results of our data reduction techniques. These variables were removed along with ones that were colinear in nature or had a high degree of missing values that were not collected in the original surveys. Various indices were tested, though the three below were chosen because their first two axes explained more than 50% of the cumulative variability.

#load libraries
library(vegan)

Loading required package: permute

Loading required package: lattice

This is vegan 2.6-6.1

library(tidyverse)

── Attaching core tidyverse packages ──────────────────────── tidyverse 2.0.0 ──
✔ dplyr 1.1.4 ✔ readr 2.1.5
✔ forcats 1.0.0 ✔ stringr 1.5.1
✔ ggplot2 3.5.1 ✔ tibble 3.2.1
✔ lubridate 1.9.3 ✔ tidyr 1.3.1
✔ purrr 1.0.2

── Conflicts ────────────────────────────────────────── tidyverse_conflicts() ──
✖ dplyr::filter() masks stats::filter()
✖ dplyr::lag() masks stats::lag()
ℹ Use the conflicted package (<http://conflicted.r-lib.org/>) to force all conflicts to become errors

library(ggplot2)
library(ggbreak)

ggbreak v0.1.2

If you use ggbreak in published research, please cite the following
paper:

S Xu, M Chen, T Feng, L Zhan, L Zhou, G Yu. Use ggbreak to effectively
utilize plotting space to deal with large datasets and outliers.
Frontiers in Genetics. 2021, 12:774846. doi: 10.3389/fgene.2021.774846

library(patchwork)
library(readxl)
library(lme4)

Loading required package: Matrix

Attaching package: 'Matrix'

The following objects are masked from 'package:tidyr':

 expand, pack, unpack

library(DHARMa)

This is DHARMa 0.4.6. For overview type '?DHARMa'. For recent changes, type news(package = 'DHARMa')

library(performance)
library(sjPlot)

Learn more about sjPlot with 'browseVignettes("sjPlot")'.

library(glmmTMB)

Warning in checkDepPackageVersion(dep_pkg = "TMB"): Package version inconsistency detected.
glmmTMB was built with TMB version 1.9.11
Current TMB version is 1.9.14
Please re-install glmmTMB from source or restore original 'TMB' package (see '?reinstalling' for more information)

library(plotrix)
library(effects)

Loading required package: carData
Use the command
 lattice::trellis.par.set(effectsTheme())
 to customize lattice options for effects plots.
See ?effectsTheme for details.

setwd("/Users/nicolescavo/Documents/Documents - ENTO-94QJ1-L/Hamer Lab/LRGV/KAPS/PLoS NTD resubmission/PLoS NTD resubmission2")

#load data
housing<-read_xlsx("Bitemark Supp Data_Clean.xlsx",sheet=2)
encuesta<-read_xlsx("Bitemark Supp Data_Clean.xlsx",sheet=3)
movement<-read_xlsx("Bitemark Supp Data_Clean.xlsx",sheet=4)
abundance<-read_xlsx("Bitemark Supp Data_Clean.xlsx",sheet=5)
#clean data - remove missing values
housing<-housing[-c(5,9,28,53,56),]
encuesta<-encuesta[-c(5,9,28,53,56),]

### Door Index

Variables related to doors and their screens (e.g., number of doors, number of doors with screens) were grouped together given their similar nature.

library(vegan)
door<-housing[,c(42, 45:48)]
pca_door<-rda(door)

**Figure A:** PCA for the door index. BAL - Balli, CHA - Chapa, IHE - Indian Hills East, IHW - Indian Hills West, MES - Mesquite, PIN - La Pinata, PRO - Progresso, SDO - South Donna. Doors with glass - number of doors with glass in them, Holes in glass - total number of holes in glass in all doors in the house, Holes in screen - total number of holes in screen in all the doors in the house, Screens - total number of doors with screens on them, Total doors - total number of doors in the house.


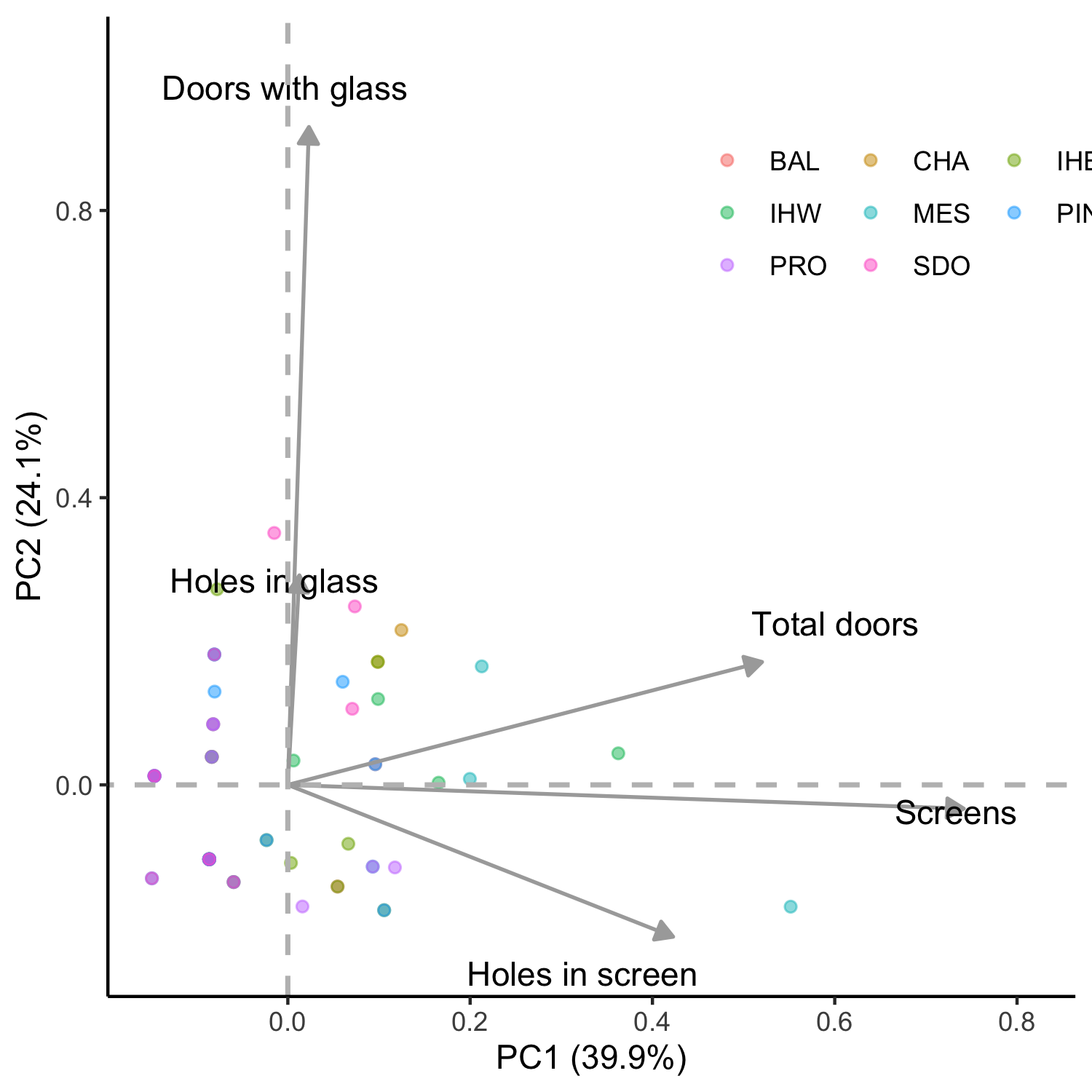


PC1 explains 39.9% and PC2 explains 24.1% of the variation in the dataset. Total number of doors, number of door screens, and number of screen with holes in them are positively associated with PC1, so, we can associate this axis with potential entry points for mosquitoes into the house. PC2 is postively associated with glass in doors.

### Window Index

Likewise, variables that were related to windows and their screens (e.g., windows with screens that have holes in them, bad screen seal).

window<-housing[,c(24:25,33:35, 37:40)]
pca_window<-rda(window)

**Figure B:** PCA for window index. BAL - Balli, CHA - Chapa, IHE - Indian Hills East, IHW - Indian Hills West, MES - Mesquite, PIN - La Pinata, PRO - Progresso, SDO - South Donna. AC bad seals - number of AC units that a bad seal with gaps allowing mosquitoes to enter the house, AC units - total number of AC units on the house, Glass with holes - number of holes in the glass in all the windows in the house, Open screens - number of screens that are able to be opened, Open windows - number of windows that are able to be opened, Screens with bad seal - number of window screen that have a bad seal allowing mosquitoes to enter the house, Screens with holes - total number of holes in all the window screens in the house, Total screens - total number of screens on windows in the house, Total windows - total number of windows in the house.


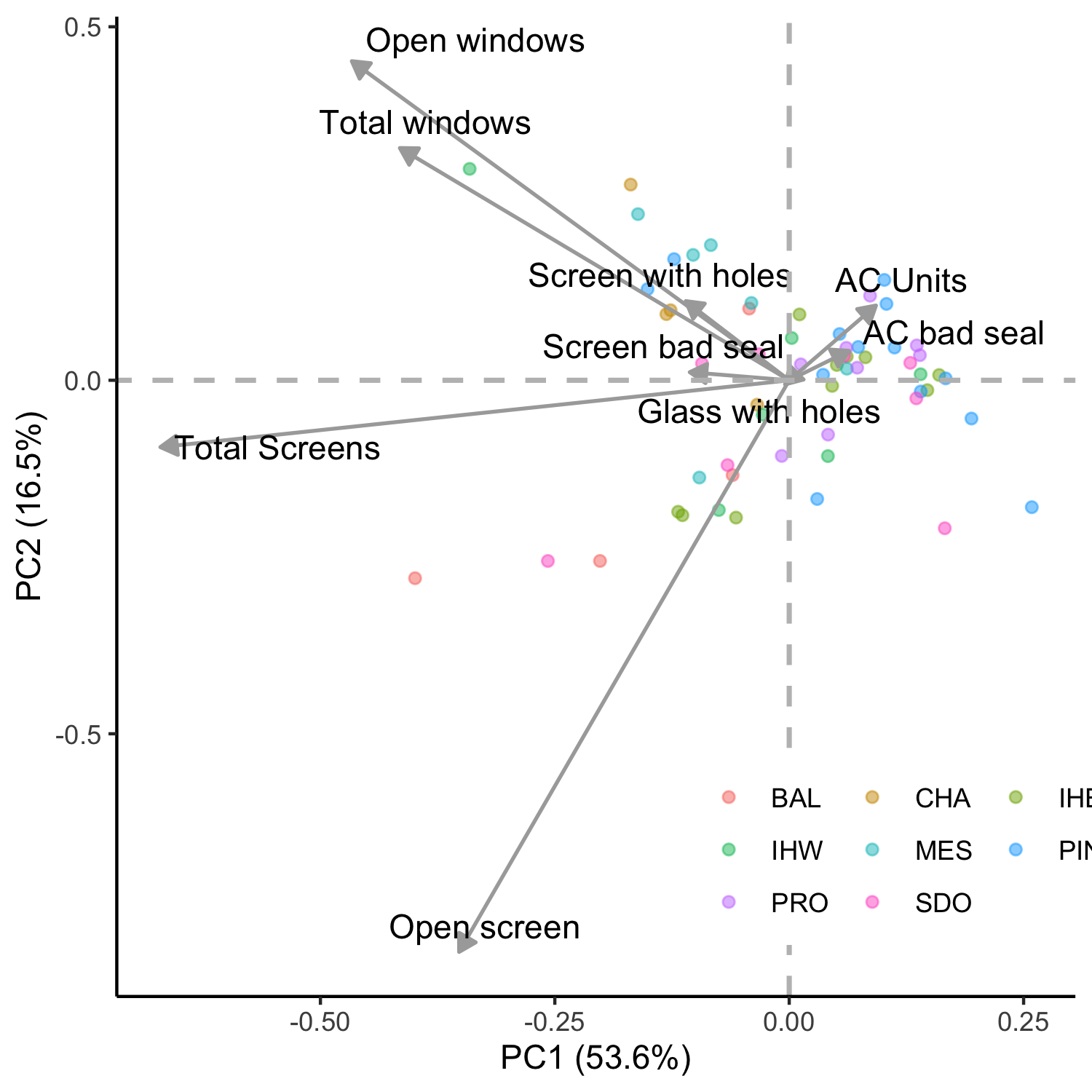


PC1 explains 53.6% of the variation in the dataset and is positively related to AC units and negatively related to the number of windows and screens in the house. PC2 explains 16.5% of the variation and is related to entry points for mosquitoes into the house. Negative values represent entry points via windows or screens and positive values represent entry points via the AC units.

### Host Index

The host index was created by grouping variables related to vertebrate hosts present in household or yard (e.g., number of dogs, number of humans within an age bracket).

host<-encuesta[11:15]
pca_host<-rda(host)

**Figure C:** PCA for host index. BAL - Balli, CHA - Chapa, IHE - Indian Hills East, IHW - Indian Hills West, MES - Mesquite, PIN - La Pinata, PRO - Progresso, SDO - South Donna. Below 5 - number of humans in the household below the age of 5, 5 to 18 - number of humans in the house hold between the ages of 5 and 18, Over 18 - number of humans in the household over the age of 18, Cats - number of cats in the household, Dogs - number of dogs in the household.


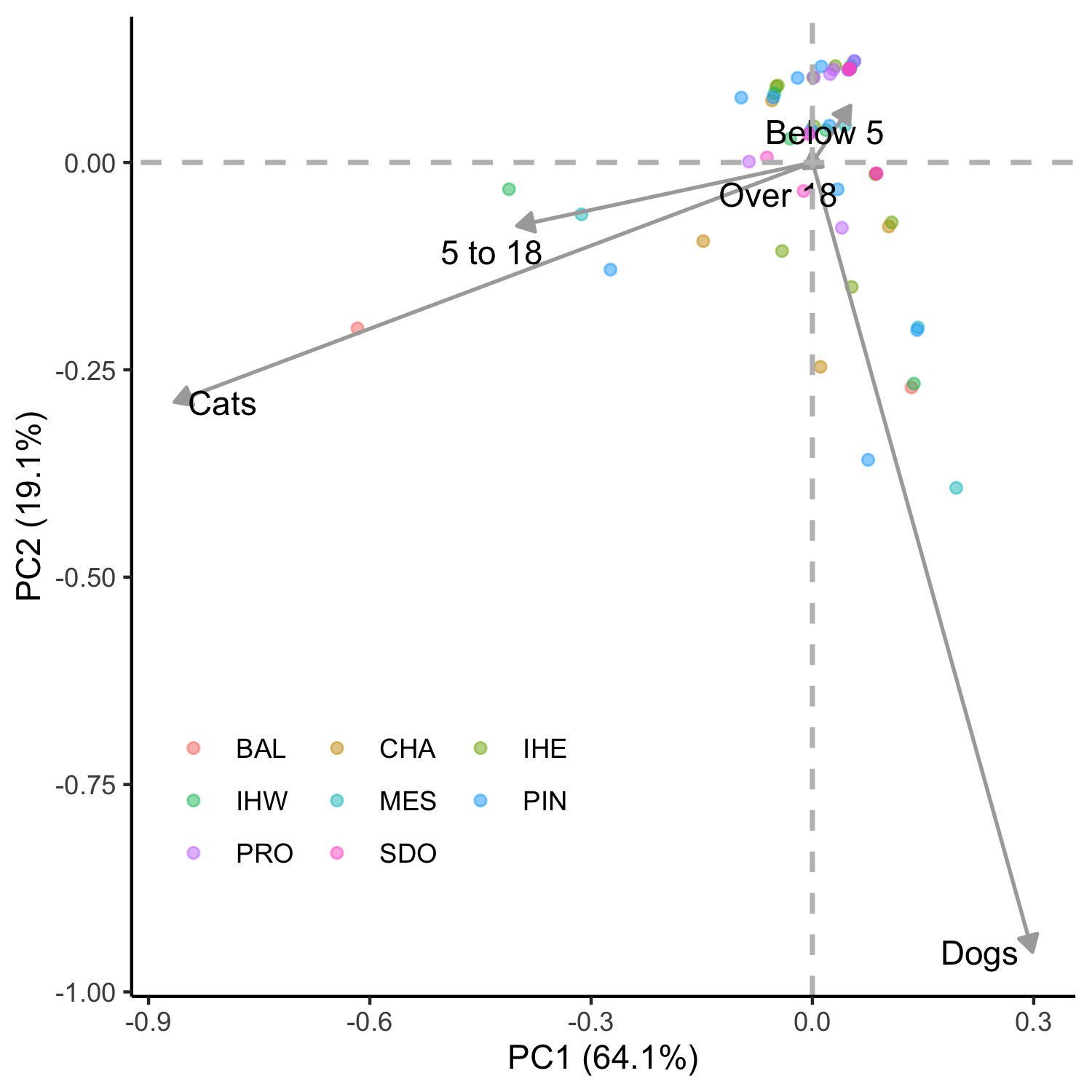


The first axis of the Host PCA explains 64.1% of the variation and is negatively associated with cats and number of children between the ages of 5 and 18. The second axis explains 19.1% of the variation is negatively associated with dogs.

## Data summary

### Explanatory Variables

After data reduction and the elimination of logistical variables (e.g., street names, latitude) from the dataset, we identified 10 variables that were relevant to our study based on our knowledge of the literature. We chose to include 10 variables to have a *n/k* ratio of at least 10, where *n* is the number of data points and *k* is the number of parameters. Finally, we standardized the continuous variables so each had a mean of zero and a standard deviation of 1. This was done since the explanatory variables were on different scales or order of magnitudes which can cause issues in modelling efforts.

#Create dataframe to use for GLMM
door.1<-pca_door$CA$u[,1]
door.2<-pca_door$CA$u[,2]
window.1<-pca_window$CA$u[,1]
window.2<-pca_window$CA$u[,2]
host.1<-pca_host$CA$u[,1]
host.2<-pca_host$CA$u[,2]
indices<-cbind(door.1, door.2, window.1, window.2, host.1, host.2)
indices<-as.data.frame(indices)
merged<-cbind(encuesta,indices)
merged2<-merge(merged, housing, by = "KAPS_ID")
merged3<-merge(merged2, movement, by = "KAPS_ID")
risk<-merge(merged3, abundance, by = "KAPS_ID")

#Transform characters to factors
risk2<-risk %>% mutate_if(is.character, as.factor)

#Select relevant variables
vars_to_keep<- c(82,1,72,74:81,83:95,17,19:24,27:31,4,33,37,46,5)
risk3<- risk2[, vars_to_keep]

#standardize continuous variables to have a mean of zero and standard deviation of 1
columns_to_stanardize<- c("Age", "Area", "door.2", "week5Avg", "ContainersTotal",
 "DistanceAverage", "host.1", "host.2")
risk4 <- risk3 %>% mutate_at(columns_to_stanardize, ~scale(.) %>% as.vector)

**Table A:** Descriptive statistics for the selected predictor variables before standardization to be used in model construction.

| Variable (unit) | Description | Options (count) | Mean | SD | Range |
| --- | --- | --- | --- | --- | --- |
| AC Type | Type of air conditioning used in the household | Central (13)  Minisplit (6)  None (3)  Window (36) |  |  |  |
| Age (years) | Age of individual participants |  | 37.2 | 22.5 | 5 - 86 |
| Area (m²) | Area of the yard |  | 688 | 249 | 369 - 1521 |
| Containers Total | Number of containers suitable as larval habitat in the yard |  | 8.3 | 9.5 | 0 - 40 |
| Distance Average (mi) | Average distance an indivdual travels in a week |  | 12.2 | 57.5 | 0 - 600 |
| door.2 | PC2 from door index |  | 0.0 | 0.1 | -0.2 - 0.4 |
| host.1 | PC1 from host index |  | 0.0 | 0.1 | -0.6 - 0.1 |
| host.2 | PC2 from host index |  | 0.0 | 0.1 | -0.4 - 0.1 |
| Income | Annual household income | < $25,000 (84)  > $25,000 (45) |  |  |  |
| Orderliness | The level of organization (e.g., amount of trash, upkeep) in the yard | Disorderly (24)  Average (20)  Orderly (10) |  |  |  |
| Sex | Sex of the individual | Female (79)  Male (50) |  |  |  |
| Vegetation Level | Percentage of yard that is covered in vegetation | <25% (17)  25-50% (12)  51-75% (18)  >75% (7) |  |  |  |
| Water Storage | If water was stored in the yard | Yes (7)  No (46) |  |  |  |
| Week 5 Average | Female *Ae. aegypti* caught per trap night averaged over 5 weeks prior to blood sampling |  | 3.4 | 2.9 | 0 - 14 |

### Outcome Variables

The outcome variable for exposure to *Ae. aegypti* bites, the Bitemark assay, is measured in 𝚫OD and is continuous.

**Figure D.** Histogram of 𝚫OD.


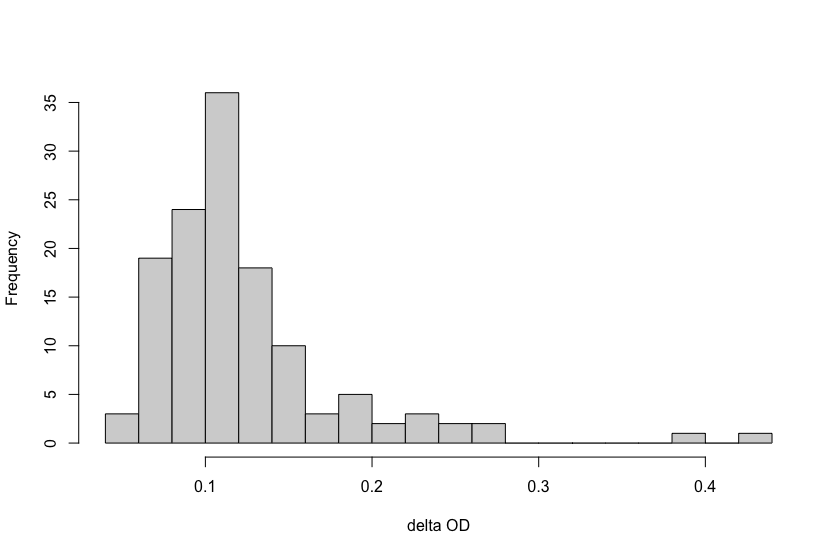


## Model Construction - Bitemark Assay

### Global Model

We built a global mixed model (mglobal1B) to evaluate the effects of the selected 10 fixed effects on 𝚫OD while controlling for the non-independence among individuals surveyed in the same houses and houses surveyed in the same communities. Given that our outcome variable, 𝚫OD, is continuous, we started with a Gaussian distribution with the identity link.

mglobal1B<-glmmTMB(N34kDa ~ ACType+Income+Age+Area+door.2+week5Avg+Sex+ContainersTotal+DistanceAverage+host.2+(1|Comm_ID:BG_ID:Person_ID),data=risk4, family = gaussian(link = "identity"), na.action = "na.omit")

We checked the error distribution using the simulateResiduals function to assess the QQ plot of mglobal1B. These plots indicated that the residuals were not normally distributed so this model was not a good choice to use in the analysis.

**Figure E.** DHARMa residual plots for mglobal1B.


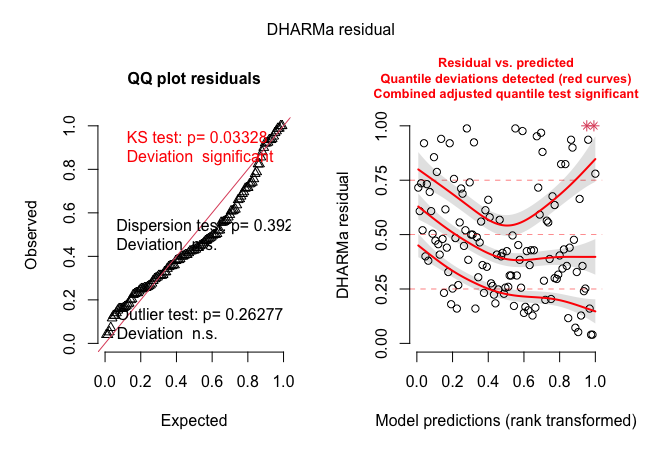


Because of these results, we constructed a second global mixed effects model (mglobal2B) with a Gaussian distribution with a log link.

mglobal2B<-glmmTMB(N34kDa ~ ACType+Income+Age+Area+door.2+week5Avg+Sex+ContainersTotal+DistanceAverage+host.2+(1|Comm_ID:BG_ID:Person_ID),
data=risk4, family = gaussian(link = "log"), na.action = "na.omit")

Using the same methods described for mglobal1B, we made a QQ plot to assess the residual distribution (Figure F). The results of which showed a normal distribution of residuals, so we used mgloabl2B as the basis for our model selection techniques.

**Figure F:** DHARMa residual plots for mglobal2B.


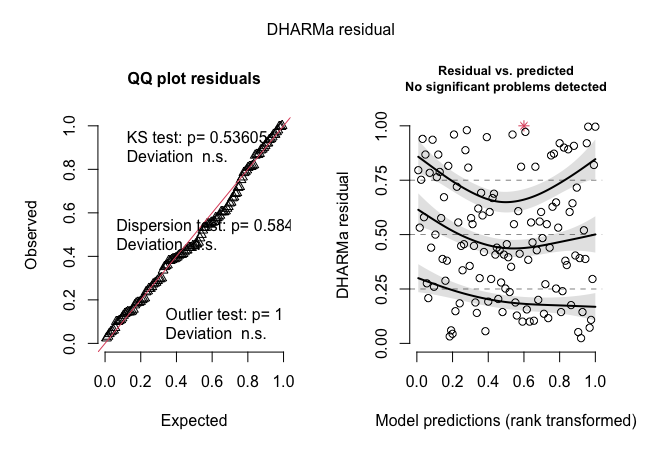


### Model Reduction

We used backward elimination to simplify the mglobal2B eliminating parameters based on the significance of their fixed effect estimates (Table B).

m1B<-glmmTMB(N34kDa ~ ACType+Income+Age+Area+week5Avg+Sex+
 ContainersTotal+DistanceAverage+host.2+(1|Comm_ID:BG_ID:Person_ID),
 data=risk4, family = gaussian(link = "log"), na.action = "na.omit")
m2B<-glmmTMB(N34kDa ~ Income+Age+Area+week5Avg+Sex+
 ContainersTotal+DistanceAverage+host.2+(1|Comm_ID:BG_ID:Person_ID),
 data=risk4, family = gaussian(link = "log"), na.action = "na.omit")
m3B<-glmmTMB(N34kDa ~ Income+Age+Area+week5Avg+Sex+
 ContainersTotal+DistanceAverage+(1|Comm_ID:BG_ID:Person_ID),
 data=risk4, family = gaussian(link = "log"), na.action = "na.omit")
m4B<-glmmTMB(N34kDa ~ Income+Age+Area+week5Avg+Sex+
 ContainersTotal+(1|Comm_ID:BG_ID:Person_ID),
 data=risk4, family = gaussian(link = "log"), na.action = "na.omit")
m5B<-glmmTMB(N34kDa ~ Income+Age+Area+week5Avg+
 ContainersTotal+(1|Comm_ID:BG_ID:Person_ID),
 data=risk4, family = gaussian(link = "log"), na.action = "na.omit")
m6B<-glmmTMB(N34kDa ~ Income+Age+Area+week5Avg+
 (1|Comm_ID:BG_ID:Person_ID),
 data=risk4, family = gaussian(link = "log"), na.action = "na.omit")

**Table B.** 𝚫AICc comparison of mglobal2B and m1B-m6B.

| Model | AIC | 𝚫AIC |
| --- | --- | --- |
| Mglobal2B | -426.8 | 17.0 |
| m1B | -428.8 | 15.0 |
| m2B | -438.4 | 5.4 |
| m3B | -440.3 | 3.5 |
| m4B | -442.2 | 1.6 |
| m5B | -443.4 | 0.4 |
| m6B | -443.8 | 0.0 |

The best fit model was m6B, though m4B and m5B are competing models as their 𝚫AIC is <2. We ran an ANOVA to find the most parsimonious model, though m4 and m5 did not explain significantly more variation than the simpler model (m6B; Table C). This model observed that income, participant age, yard area, and the average number of female *Ae. aegypti* per trap night had significant effects on 𝚫OD (Table D).

**Table C.** Results of ANOVA comparing the top three models that have a 𝚫AICc of <2.

|  | df | AIC | BIC | logLik | deviance | Chisq | Chi Df | Pr(>Chisq) |
| --- | --- | --- | --- | --- | --- | --- | --- | --- |
| m4B | 9 | -442.17 | -416.64 | 228.91 | -457.83 | 0.73 | 1 | 0.39 |
| m5B | 8 | -443.44 | -420.75 | 229.72 | -459.44 | 1.61 | 1 | 0.20 |
| m6B | 7 | -443.83 | -423.97 | 230.08 | -460.17 |  |  |  |

**Table D.** Main effects of the GLMM m6B for risk factors for exposure to *Aedes aegypti* bites. Bolded *p* values are statistically significant.

| Predictors | Exponentiaed Estimate | Estimates | 95% CI (Exponentiated) | *p* |
| --- | --- | --- | --- | --- |
| Intercept |  | -2.24 |  | **<0.001** |
| Income (>$25,000) | 1.21 | 0.19 | 0.07-0.32 | **0.003** |
| Age | 0.93 | -0.07 | -0.13 - -0.01 | **0.032** |
| Area | 1.11 | 0.10 | 0.04 - 0.16 | **0.002** |
| week5Avg | 1.12 | 0.11 | 0.05 - 0.17 | **0.001** |

## Model Construction - Adult Female *Ae. aegypti* abundance

### Global Models

We built three global mixed models to evaluate the effects 10 fixed effects on the outdoor abundance of adult female *Ae. aegypti* in the five weeks before the blood sampling for the Bitemark Assay took place. Community and intervention arms were used as a random variables. Given that our outcome variable was count data, we tested Poisson (mgloabl1A), Negative Binomial 2 (mglobal2A), and Negative Binomial 1 (mbglobal3A) distributions. We checked for correlation among variables before modelling and removed any variables that had above a 0.8 correlation.

mglobal1A<-glmmTMB(AeaeFemaleTotal ~ offset(log(WeeksIn)) + WaterStorage + ContainersTotal +
host.1 + host.2 + Income + VegetationLevel + Area + Orderliness + PrecipCum + (1|Comm_ID) + (1|Intervention), data = risk4, family = genpois(), na.action = "na.omit")

mglobal2A<-glmmTMB(AeaeFemaleTotal ~ offset(log(WeeksIn)) + WaterStorage + ContainersTotal +
host.1 + host.2 + Income + VegetationLevel + Area + Orderliness + PrecipCum + (1|Comm_ID) + (1|Intervention), data = risk4, family = nbinom2(), na.action = "na.omit")

mglobal3A<-glmmTMB(AeaeFemaleTotal ~ offset(log(WeeksIn)) + WaterStorage + ContainersTotal + host.1 + host.2 + Income + VegetationLevel + Area + Orderliness + PrecipCum + (1|Comm_ID) + (1|Intervention), data = risk4, family = nbinom1(), na.action = "na.omit")

We then compared models using AIC values, assessed the fit of the model using QQ plots, and checked for multicollinearity. mgloabl2A had the lowest AIC value at 867.9 making it the best fit model.

**Figure G.** DHARMa residual plots for mglobal2A.


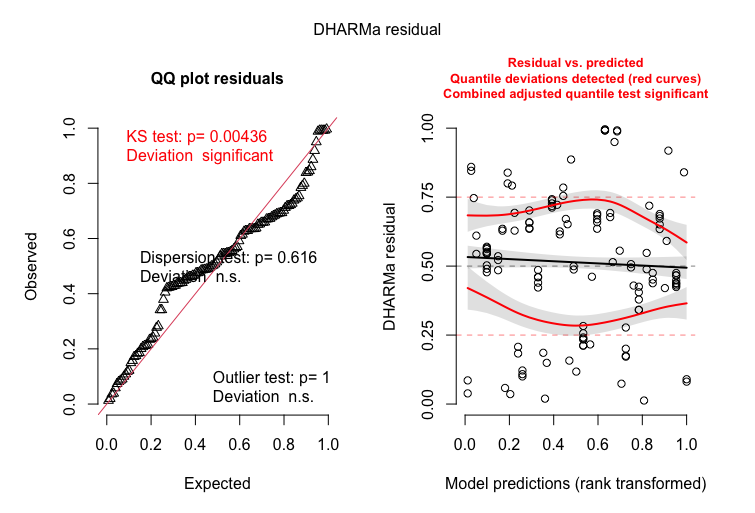


### Model Reduction

We used backward elimination to simplify the mglobal2A eliminating parameters based on the significance of their fixed effect estimates.

m1A<-glmmTMB(AeaeFemaleTotal ~ offset(log(WeeksIn)) + WaterStorage + ContainersTotal + host.2 + Income + VegetationLevel + Area + Orderliness + PrecipCum + (1|Comm_ID) + (1|Intervention), data = risk4, family = nbinom2(), na.action = "na.omit")

m2A<-glmmTMB(AeaeFemaleTotal ~ offset(log(WeeksIn)) + WaterStorage + ContainersTotal + host.2 + Income + VegetationLevel + Area + PrecipCum + (1|Comm_ID) + (1|Intervention), data = risk4, family = nbinom2(), na.action = "na.omit")

m3A<-glmmTMB(AeaeFemaleTotal ~ offset(log(WeeksIn)) + ContainersTotal + host.2 + Income + VegetationLevel + Area + PrecipCum + (1|Comm_ID) + (1|Intervention), data = risk4, family = nbinom2(), na.action = "na.omit")

m4A<-glmmTMB(AeaeFemaleTotal ~ offset(log(WeeksIn)) + ContainersTotal + host.2 + Income + VegetationLevel + Area + (1|Comm_ID) + (1|Intervention), data = risk4, family = nbinom2(),
na.action = "na.omit")

m5A<-glmmTMB(AeaeFemaleTotal ~ offset(log(WeeksIn)) + ContainersTotal + Income + VegetationLevel + Area + (1|Comm_ID) + (1|Intervention), data = risk4, family = nbinom2(), na.action = "na.omit")

m6A<-glmmTMB(AeaeFemaleTotal ~ offset(log(WeeksIn)) + ContainersTotal + Income + Area + (1|Comm_ID) + (1|Intervention), data = risk4, family = nbinom2(), na.action = "na.omit")

**Table D.** 𝚫AICc comparison of m1A-m6A.

| Model | AIC | 𝚫AIC |
| --- | --- | --- |
| m1A | 865.9 | 1.7 |
| m2A | 864.2 | 0.0 |
| m3A | 869.1 | 4.9 |
| m4A | 869.2 | 5.0 |
| m5A | 869.5 | 5.3 |
| m6A | 871.9 | 7.4 |

The best fit model was m2A, though m1A was a competing model with a 𝚫AIC of 1.7 (Table D). An ANOVA showed that m1A did not explain significantly more variation than m2A (Table E). m2A had seven explanatory variables, four of which were significant: total number of containers in the yard, income, medium vegetation levels, and area of yard (Table F).

**Table E.** Results of ANOVA comparing the top two models that have a 𝚫AICc of <2.

|  | df | AIC | BIC | logLik | deviance | Chisq | Chi Df | Pr(>Chisq) |
| --- | --- | --- | --- | --- | --- | --- | --- | --- |
| m1A | 13 | 864.2 | 901.3 | -419.1 | 838.2 | 2.30 | 2 | 0.32 |
| m2A | 15 | 865.9 | 908.7 | -418.0 | 835.9 |  |  |  |

**Table F.** Main effects of the GLMM m2A for risk factors for outdoor *Aedes aegypti* female abundance. Marginal R² = 0.12, Conditional R² = 0.54. Bolded *p* values are statistically significant.

| Predictor | Exponentiated Estimate | Estimate | 95% CI (exponentiated) | *p* |
| --- | --- | --- | --- | --- |
| Intercept |  | 1.00 |  | **<0.001** |
| WaterStorage (Yes) | 0.89 | -0.12 | 0.68 - 1.16 | 0.388 |
| ContainersTotal | 1.16 | 0.15 | 1.06 - 1.27 | **0.002** |
| host.2 | 1.06 | 0.06 | 0.98 - 1.15 | 0.127 |
| Income (>$25,000) | 0.82 | -0.20 | 0.68 - 0.98 | **0.028** |
| VegeationLevel (>75%) | 1.03 | 0.03 | 0.76 - 1.41 | 0.849 |
| VegeationLevel (>25-50%) | 1.20 | 0.18 | 1.00 - 1.44 | **0.045** |
| VegeationLevel (>51-75%) | 0.87 | -0.14 | 0.69 - 1.09 | 0.225 |
| Area | 0.82 | -0.20 | 0.74 - 0.90 | **<0.001** |
| PrecipCum | 1.23 | 0.21 | 0.88 - 1.72 | 0.233 |
