# Supplementary material for: Little disease but lots of bites: social, urbanistic, and entomological risk factors of human exposure to Aedes aegypti in South Texas, U.S
Source: PLoS Negl Trop Dis. 2024 Oct 21;18(10):e0011953. doi: 10.1371/journal.pntd.0011953 (PMC11527178; doi:10.1371/journal.pntd.0011953)
Supplement: S1 Code — (RTF) [file pntd.0011953.s002.rtf]

#R code for Bitemark Analysis #Load libraries library(vegan) library(tidyverse) library(ggplot2) library(ggrepel) library(ade4) library(FactoMineR) library(psych)library(DHARMa)library(spdep)library(ape)library(car)library(performance)library(sjPlot)library(effects)library(readxl)library(glmmTMB)#Load datahousing<-read_xlsx("S1_Data_revised.xlsx",sheet=2)encuesta<-read_xlsx("S1_Data_revised.xlsx",sheet=3)movement<-read_xlsx("S1_Data_revised.xlsx",sheet=4)abundance<-read_xlsx("S1_Data_revised.xlsx",sheet=5)#Clean data (remove rows with missing values)housing<-housing[-c(5,9,28,53,56),]encuesta<-encuesta[-c(5,9,28,53,56),]#Information for tables in manuscript table(encuesta$WaterStorage)table(encuesta$ACType)table(housing$VegetationLevel)table(housing$VegLength)table(housing$ShadeCover)table(housing$Orderliness)table(housing$HouseType)table(housing$RoofMaterial)table(housing$WallMaterial)table(housing$TotalRooms)#Create door indexdoor<-housing[,c(42, 45:48)]pca_door<-rda(door)summary(pca_door)ordiplot(pca_door, display = "species", type = "text", xlab = "PC1 (39.9%)", ylab = "PC2 (24.1%)", main = "Door Index")#Figure of PCA for publicationpca_door_plot<-ggplot() +  geom_point(data = as.data.frame(pca_door$CA$u[,1:2]),             aes(x = pca_door$CA$u[,1], y = pca_door$CA$u[,2],                  colour=housing$Comm_code),             alpha=0.5) +  geom_segment(data = as.data.frame(pca_door$CA$v),               mapping = aes(x = 0, y = 0, xend = pca_door$CA$v[,1], yend = pca_door$CA$v[,2]),               arrow = arrow(length = unit(0.02, "npc"),                             type="closed"),               colour="darkgray",               size =0.6) +  geom_text_repel(data = as.data.frame(pca_door$CA$v[,1:2]), # crudely push labels away arrow heads                  mapping = aes(label = c("Total doors",                                           "Doors with glass", "Holes in glass", "Screens",                                          "Holes in screen"),                                 x = pca_door$CA$v[,1] * 1.1, y = pca_door$CA$v[,2] * 1.1)) +  geom_abline(intercept = 0,slope = 0,linetype="dashed", linewidth=0.8,colour="gray") +  geom_vline(aes(xintercept=0), linetype="dashed", linewidth=0.8,colour="gray") +  xlab("PC1 (39.9%)") +  ylab("PC2 (24.1%)") +  theme(panel.grid.major = element_blank(),        panel.grid.minor = element_blank(),        panel.background = element_blank(),        axis.line = element_line(colour = "black"),        legend.position = c(0.82,0.8),        legend.background = element_rect()) +  guides(color=guide_legend(nrow=3, byrow=T, title = NULL)) pca_door_plotpng("Door PCA.png", width = 5, height = 5, units = "in",     res = 300)pca_door_plotdev.off()# Create window index window<-housing[,c(24:25,33:35, 37:40)]pca_window<-rda(window)summary(pca_window)ordiplot(pca_window, display = "species", type = "text", xlab = "PC1 (53.6%)",         ylab = "PC2 (16.5%)", main = "Window Index")#Make figure for publication pca_window_plot<-ggplot() +  geom_point(data = as.data.frame(pca_window$CA$u[,1:2]),             aes(x = pca_window$CA$u[,1], y = pca_window$CA$u[,2],                  colour=housing$Comm_code),             alpha=0.5) +  geom_segment(data = as.data.frame(pca_window$CA$v),               mapping = aes(x = 0, y = 0, xend = pca_window$CA$v[,1], yend = pca_window$CA$v[,2]),               arrow = arrow(length = unit(0.02, "npc"),                             type="closed"),               colour="darkgray",               size =0.6) +  geom_text_repel(data = as.data.frame(pca_window$CA$v[,1:2]),                   mapping = aes(label = c("Total windows", "Open windows", "Glass with holes",                                           "Total Screens",                                           "Open screen","Screen with holes",                                           "Screen bad seal","AC Units",                                            "AC bad seal"),                                 x = pca_window$CA$v[,1], y = pca_window$CA$v[,2])) +  geom_abline(intercept = 0,slope = 0,linetype="dashed", linewidth=0.8,colour="gray") +  geom_vline(aes(xintercept=0), linetype="dashed", linewidth=0.8,colour="gray") +  xlab("PC1 (53.6%)") +  ylab("PC2 (16.5%)") +  theme(panel.grid.major = element_blank(),        panel.grid.minor = element_blank(),        panel.background = element_blank(),        axis.line = element_line(colour = "black"),        legend.position = c(0.82,0.15),        legend.background = element_rect()) +  guides(color=guide_legend(nrow=3, byrow=T, title = NULL))pca_window_plotpng("Window PCA.png", width = 5, height = 5, units = "in",     res = 300)pca_window_plotdev.off()#Create host index host<-encuesta[9:13]pca_host<-rda(host)summary(pca_host)ordiplot(pca_host, display = "species", type  = "text", xlab = "PC1 (64.1%)", ylab = "PC2 (19.1%)")#Make figure for publicationpca_host_plot<-ggplot() +  geom_point(data = as.data.frame(pca_host$CA$u[,1:2]),             aes(x = pca_host$CA$u[,1], y = pca_host$CA$u[,2],                  colour=encuesta$Comm_code),             alpha=0.5) +  geom_segment(data = as.data.frame(pca_host$CA$v),               mapping = aes(x = 0, y = 0, xend = pca_host$CA$v[,1], yend = pca_host$CA$v[,2]),               arrow = arrow(length = unit(0.02, "npc"),                             type="closed"),               colour="darkgray",               size =0.6) +  geom_text_repel(data = as.data.frame(pca_host$CA$v[,1:2]), # crudely push labels away arrow heads                  mapping = aes(label = c("Over 18", "Below 5", "5 to 18", "Dogs", "Cats"),                                 x = pca_host$CA$v[,1], y = pca_host$CA$v[,2])) +  geom_abline(intercept = 0,slope = 0,linetype="dashed", linewidth=0.8,colour="gray") +  geom_vline(aes(xintercept=0), linetype="dashed", linewidth=0.8,colour="gray") +  xlab("PC1 (64.1%)") +  ylab("PC2 (19.1%)") +  theme(panel.grid.major = element_blank(),        panel.grid.minor = element_blank(),        panel.background = element_blank(),        axis.line = element_line(colour = "black"),        legend.position = c(0.25,0.2),        legend.background = element_rect()) +  guides(color=guide_legend(nrow=3, byrow=T, title = NULL))pca_host_plotpng("Host PCA.png", width = 5, height = 5, units = "in",     res = 300)pca_host_plotdev.off()#Create dataframe to use for GLMMdoor.1<-pca_door$CA$u[,1]door.2<-pca_door$CA$u[,2]window.1<-pca_window$CA$u[,1]window.2<-pca_window$CA$u[,2]host.1<-pca_host$CA$u[,1]host.2<-pca_host$CA$u[,2]indices<-cbind(door.1, door.2, window.1, window.2, host.1, host.2)indices<-as.data.frame(indices)merged<-cbind(encuesta,indices)merged2<-merge(merged, housing, by = "KAPS_ID")merged3<-merge(merged2, movement, by = "KAPS_ID")risk<-merge(merged3, abundance, by = "KAPS_ID")#Transform characters to factorsrisk2<-risk %>% mutate_if(is.character, as.factor)summary(risk2)#Select relevant variables vars_to_keep<- c(82,1,72,74:81,83:95,17,19:24,27:31,4,33,37,46,5)risk3<- risk2[, vars_to_keep]#standardize continuous variables to have a mean of zero and standard deviation of 1 columns_to_stanardize<- c("Age", "Area", "door.2", "week5Avg", "ContainersTotal",                           "DistanceAverage", "host.1", "host.2")risk4 <- risk3 %>% mutate_at(columns_to_stanardize, ~scale(.) %>% as.vector)#make categorical variables numerics for later correlation checksrisk4_numeric <- risk4 %>%  mutate(across(where(is.factor), ~ as.numeric(as.factor(.))))#Modeling - Bitemark#check for correlation among vairables in global modelsprint(cor(risk4_numeric[c(41,25,5,27,23,4,40,9,31)]))#global model 1 - Gaussian with identity link mglobal1B<-glmmTMB(N34kDa ~ ACType+Income+Age+Area+door.2+week5Avg+Sex+                 ContainersTotal+DistanceAverage+host.2+(1|Comm_ID:BG_ID:Person_ID),               data=risk4, family = gaussian(link = "identity"), na.action = "na.omit")summary(mglobal1B)tab_model(mglobal1B)mglobal1B_V<- simulateResiduals(fittedModel = mglobal1B, n = 250,                                plot = TRUE)#global model 2 - Gaussian with log linkmglobal2B<-glmmTMB(N34kDa ~ ACType+Income+Age+Area+door.2+week5Avg+Sex+                    ContainersTotal+DistanceAverage+host.2+(1|Comm_ID:BG_ID:Person_ID),                  data=risk4, family = gaussian(link = "log"), na.action = "na.omit")summary(mglobal2B)tab_model(mglobal2B)mglobal2B_V<- simulateResiduals(fittedModel = mglobal2B, n = 250,                                plot = TRUE)#Backward elimination m1B<-glmmTMB(N34kDa ~ ACType+Income+Age+Area+week5Avg+Sex+                    ContainersTotal+DistanceAverage+host.2+(1|Comm_ID:BG_ID:Person_ID),                  data=risk4, family = gaussian(link = "log"), na.action = "na.omit")summary(m1B)tab_model(m1B)m1B_V<- simulateResiduals(fittedModel = m1B, n = 250,                                plot = TRUE)m2B<-glmmTMB(N34kDa ~ Income+Age+Area+week5Avg+Sex+              ContainersTotal+DistanceAverage+host.2+(1|Comm_ID:BG_ID:Person_ID),            data=risk4, family = gaussian(link = "log"), na.action = "na.omit")summary(m2B)tab_model(m2B)m2B_V<- simulateResiduals(fittedModel = m2B, n = 250,                          plot = TRUE)m3B<-glmmTMB(N34kDa ~ Income+Age+Area+week5Avg+Sex+              ContainersTotal+DistanceAverage+(1|Comm_ID:BG_ID:Person_ID),            data=risk4, family = gaussian(link = "log"), na.action = "na.omit")summary(m3B)tab_model(m3B)m3B_V<- simulateResiduals(fittedModel = m3B, n = 250,                          plot = TRUE)m4B<-glmmTMB(N34kDa ~ Income+Age+Area+week5Avg+Sex+              ContainersTotal+(1|Comm_ID:BG_ID:Person_ID),            data=risk4, family = gaussian(link = "log"), na.action = "na.omit")summary(m4B)tab_model(m4B)m4B_V<- simulateResiduals(fittedModel = m4B, n = 250,                          plot = TRUE)m5B<-glmmTMB(N34kDa ~ Income+Age+Area+week5Avg+              ContainersTotal+(1|Comm_ID:BG_ID:Person_ID),            data=risk4, family = gaussian(link = "log"), na.action = "na.omit")summary(m5B)tab_model(m5B)m5B_V<- simulateResiduals(fittedModel = m5B, n = 250,                          plot = TRUE)m6B<-glmmTMB(N34kDa ~ Income+Age+Area+week5Avg+              (1|Comm_ID:BG_ID:Person_ID),            data=risk4, family = gaussian(link = "log"), na.action = "na.omit")summary(m6B)tab_model(m6B, show.r2 = T)m6B_V<- simulateResiduals(fittedModel = m6B, n = 250,                          plot = TRUE)#compare AICAIC(mglobal2B)AIC(m1B)AIC(m2B)AIC(m3B)AIC(m4B)AIC(m5B)AIC(m6B)anova(m4B, m5B, m6B)#make effect plots for m6tiff("alleffects,6B.tiff", width = 5, height = 5, units = "in",     res = 300)plot(allEffects(m6B))dev.off()#Modeling - Female abundance#check for correlation among vairables in global modelsprint(cor(risk4_numeric[c(37,40,30:31,25,38,36,39,34,32)]))#global model 1A - poissonmglobal1A<-glmmTMB(AeaeFemaleTotal ~ offset(log(WeeksIn)) + WaterStorage + ContainersTotal +                     host.1 + host.2 + Income + VegetationLevel + Area + Orderliness +                     PrecipCum + (1|Comm_ID) + (1|Intervention), data = risk4, family = genpois(),                    na.action = "na.omit")summary(mglobal1A)tab_model(mglobal1A)mglobal1A_V<- simulateResiduals(fittedModel = mglobal1A, n = 250,                                plot = TRUE)#global model 2A - negative bionomial 2mglobal2A<-glmmTMB(AeaeFemaleTotal ~ offset(log(WeeksIn)) + WaterStorage + ContainersTotal +                     host.1 + host.2 + Income + VegetationLevel + Area + Orderliness +                     PrecipCum + (1|Comm_ID) + (1|Intervention), data = risk4, family = nbinom2(),                    na.action = "na.omit")summary(mglobal2A)tab_model(mglobal2A)mglobal2A_V<- simulateResiduals(fittedModel = mglobal2A, n = 250,                                plot = TRUE)#global model 3A - negative bionomial 1mglobal3A<-glmmTMB(AeaeFemaleTotal ~ offset(log(WeeksIn)) + WaterStorage + ContainersTotal +                     host.1 + host.2 + Income + VegetationLevel + Area + Orderliness +                     PrecipCum  + (1|Comm_ID) + (1|Intervention), data = risk4, family = nbinom1(),                    na.action = "na.omit")summary(mglobal3A)tab_model(mglobal3A)mglobal3A_V<- simulateResiduals(fittedModel = mglobal3A, n = 250,                                 plot = TRUE)#compare AIC valuesAIC(mglobal1A)AIC(mglobal2A)AIC(mglobal3A)#Backward elimination m1A<-glmmTMB(AeaeFemaleTotal ~ offset(log(WeeksIn)) + WaterStorage + ContainersTotal +                      host.2 + Income + VegetationLevel + Area + Orderliness +                     PrecipCum + (1|Comm_ID) + (1|Intervention), data = risk4, family = nbinom2(),                    na.action = "na.omit")summary(m1A)m2A<-glmmTMB(AeaeFemaleTotal ~ offset(log(WeeksIn)) + WaterStorage + ContainersTotal +               host.2 + Income + VegetationLevel + Area +               PrecipCum + (1|Comm_ID) + (1|Intervention), data = risk4, family = nbinom2(),              na.action = "na.omit")summary(m2A)m3A<-glmmTMB(AeaeFemaleTotal ~ offset(log(WeeksIn)) + ContainersTotal +               host.2 + Income + VegetationLevel + Area +               PrecipCum + (1|Comm_ID) + (1|Intervention), data = risk4, family = nbinom2(),              na.action = "na.omit")summary(m3A)m4A<-glmmTMB(AeaeFemaleTotal ~ offset(log(WeeksIn)) + ContainersTotal +               host.2 + Income + VegetationLevel + Area +                (1|Comm_ID) + (1|Intervention), data = risk4, family = nbinom2(),              na.action = "na.omit")summary(m4A)m5A<-glmmTMB(AeaeFemaleTotal ~ offset(log(WeeksIn)) + ContainersTotal +                Income + VegetationLevel + Area +               (1|Comm_ID) + (1|Intervention), data = risk4, family = nbinom2(),              na.action = "na.omit")summary(m5A)m6A<-glmmTMB(AeaeFemaleTotal ~ offset(log(WeeksIn)) + ContainersTotal +               Income  + Area +               (1|Comm_ID) + (1|Intervention), data = risk4, family = nbinom2(),              na.action = "na.omit")summary(m6A)#compare AIC valuesAIC(m1A)AIC(m2A)AIC(m3A)AIC(m4A)AIC(m5A)AIC(m6A)#compare models within a delta AIC of 2anova(m1A, m2A)#make effect plots for m1Atiff("alleffects,m2A.tiff", width = 10, height = 10, units = "in",      res = 300)plot(allEffects(m2A))dev.off()
